# XIST/miR-139 axis regulates bleomycin (BLM)-induced extracellular matrix (ECM) and pulmonary fibrosis through β-catenin

**DOI:** 10.18632/oncotarget.18310

**Published:** 2017-05-31

**Authors:** Yichun Wang, Ying Liang, Junming Luo, Jing Nie, Huiming Yin, Qiong Chen, Jing Dong, Jixiang Zhu, Jiamei Xia, Wei Shuai

**Affiliations:** ^1^ Critical Care Medicine Department, Hunan Cancer Hospital, Changsha, China; ^2^ Department of Food Science and Engineering, Central South University of Forestry and Technology, Changsha, China; ^3^ Respiratory Medicine Department, The First Affiliated Hospital of Hunan University of Medicine, Changsha, China

**Keywords:** XIST/miR-139, pulmonary fibrosis (PF), fibroblast, β-catenin, extracellular matrix (ECM)

## Abstract

Pulmonary fibrosis (PF), characterized by the destruction of lung tissue architecture and the abnormal deposition of extracellular matrix (ECM) proteins, currently has no satisfactory treatment. In the present study, we investigated the hypothesis that XIST play a promotive role in bleomycin (BLM)-induced ECM and pulmonary fibrosis; XIST exerts its effect through miR-139 regulation. XIST expression was upregulated in lung tissues derived from BLM-induced mouse model of PF, and was positively correlated with β-catenin and ECM protein levels, respectively. LV-sh-XIST-induced XIST knockdown led to decreased PF, reduced β-catenin and ECM protein levels in lung tissues. XIST knockdown suppressed the proliferation of IMR-90 (human fibroblast) and murine lung fibroblasts (MLFCs) and ECM protein expression. Moreover, miR-139 could directly bind to XIST and the 3’UTR of β-catenin; XIST competed with β-catenin for miR-139 binding both in IMR-90 and MLFCs. In MLFCs, miR-139 inversely regulated XIST, and could partially reverse the effect of XIST on β-catenin and ECM proteins. In lung tissues of PF mice, miR-139 expression was downregulated, whereas β-catenin expression was upregulated. In conclusion, XIST exerts positive effects on BLM-induced PF through inhibiting miR-139 to promote human/mouse fibroblast proliferation and ECM proteins.

## INTRODUCTION

Pulmonary fibrosis (PF) is a devastating chronic lung condition with poor prognosis. Pulmonary fibrosis progresses insidiously and slowly, with acute exacerbation of interstitial pneumonia being a highly lethal clinical event. Furthermore, the mortality rate for pulmonary fibrosis is increasing [1, 2].

It has been reported that pulmonary fibrosis is induced by repeated epithelial cell damage and abnormal wound repair and remodeling, resulting in over-proliferation of fibroblasts and abnormal deposition of extracellular matrix (ECM) proteins, such as collagen I and α-SMA [3]. An increase in lung myofibroblasts has been suggested to have an important role in abnormal wound repair and remodeling [4]. Myofibroblasts extensively produce and secrete ECM proteins [4], and are involved in abnormal wound repair and remodeling through various mechanisms. Bleomycin (BLM) has been used to induce lung fibrosis, which mimics PF, in both mouse and rat models [5, 6].

Recently, a group of long non-coding RNAs (long ncRNAs, lncRNA), which are non-protein coding transcripts longer than 200 nucleotides [7], have been reported to play important roles in both normal development and diseases including pulmonary fibrosis [8, 9]. Among the large amount of lncRNAs, XIST has been reported to be specifically upregulated in cancers, and promote cancer cell proliferation. Through regulating miR-497/MACC1 axis in gastric cancer, XIST promotes cell growth and invasion [10]. Moreover, XIST has been reported to highly express in cystic fibrosis bronchial epithelium [11], suggesting the potential relation between XIST and fibrosis. However, there are few reports about the functional and mechanistic effect of XIST on pulmonary fibroblasts proliferation and pulmonary fibrosis.

The mechanisms by which lncRNAs exert their effect varies under different conditions, however, emerging evidences have revealed that the interaction between lncRNAs and microRNAs plays a major role [12, 13]. A previous study demonstrated that some lncRNAs can serve as a ‘sponge’ to microRNAs and prevent them from binding to mRNAs based on the competing endogenous RNA (ceRNA) hypothesis [14]. Pandolfi et al. reported that endogenous miRNA decoys have important functions in various biological processes and cell types, and ceRNAs can be found in all organisms that use miRNAs to regulate gene expression. Given the prominent functions of ceRNAs in physiology, their deregulation is a common occurrence in various diseases that can promote their progression [15, 16]. Therefore, identifying well-established miRNAs that bind lncRNAs and their potential downstream target genes may help to infer the function of lncRNAs.

According to previous studies, several signaling pathways were involved in fibrosis progression; among them Wnt/β-catenin has been reported to play an essential role [17]. Recently, emerging evidence from researches of organ fibrosis suggests that sustained Wnt/beta-catenin pathway reactivation is linked to the pathogenesis of fibrotic disorders [18–20]. Here, we investigated whether XIST exerts its function in BLM-induced PF through Wnt/β-catenin signaling.

In the present study, we constructed BLM-induced mouse model of PF, evaluated the detailed functions of XIST in PF mouse, pulmonary fibroblasts proliferation and ECM protein expression in IMR-90 and murine lung fibroblasts (MLFCs). By using online tools, we found that XIST and β-catenin share an almost identical binding site of miR-139. Through Wnt/β-catenin signaling, XIST/miR-139 regulates ECM protein expression in MLFCs. Taken together, we revealed the promotive effect of XIST on BLM-induced PF, and demonstrated the mechanism by which XIST/miR-139 axis exerts its effect on regulating pulmonary fibrosis, providing a potential therapy to treat PF.

## RESULTS

### Expression and function of XIST in BLM-induced mouse model of PF

**Figure 1:**
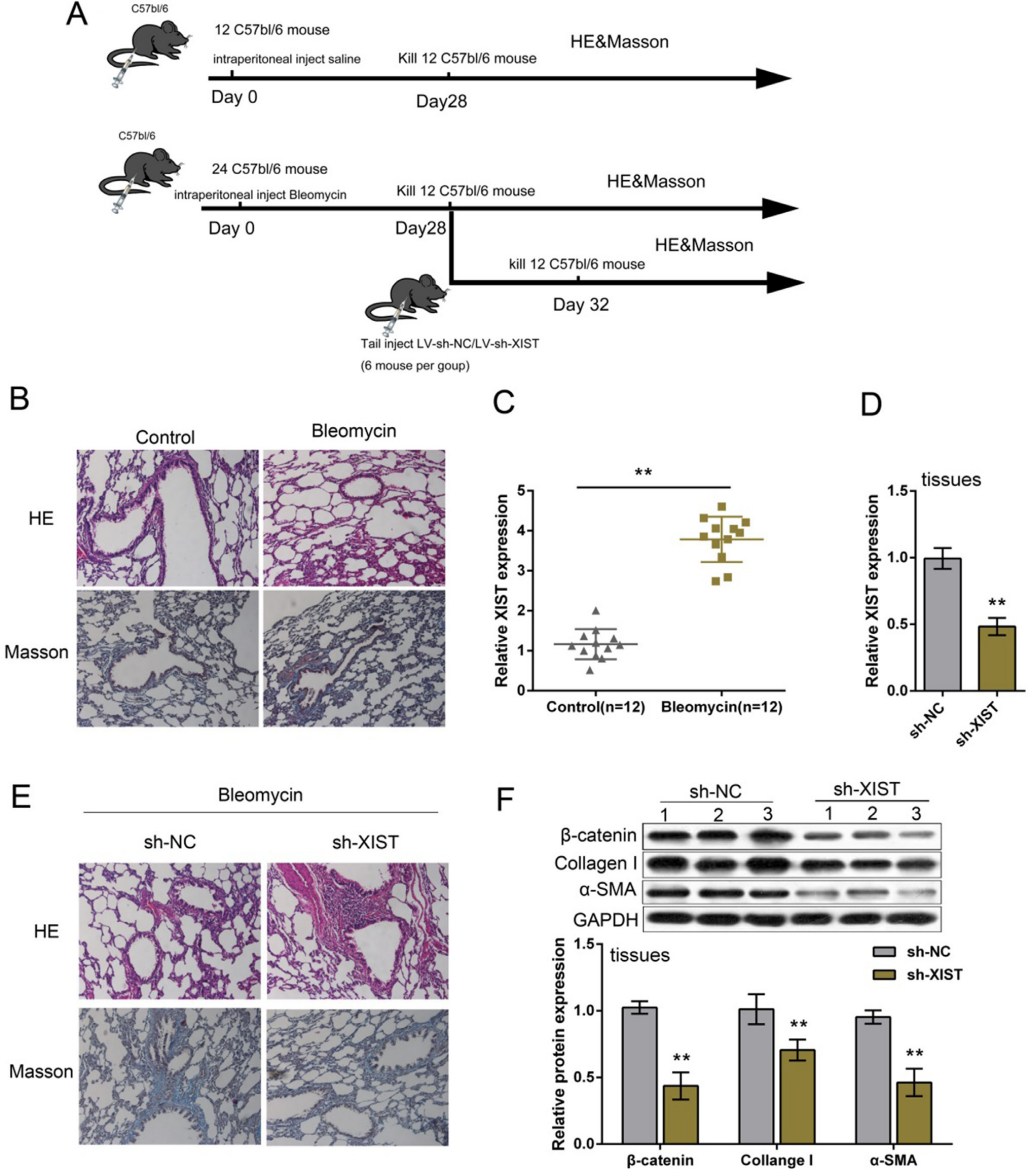
Expression and function of XIST in BLM-induced mouse model of PF (**A**) A total of 36 mice were involved in BLM-induced mouse model of PF; a group of 12 mice were injected with saline (control), sacrificed on day 28 after injection, lung tissues obtained for RNA extraction, HE staining or Masson’s trichrome assay; a group of 24 mice were injected with BLM, 12 of them sacrificed on day 28, the rest 12 of them were infected with LV-sh-NC (control) or LV-sh-XIST on day 28, sacrificed 4 more days later for RNA extraction, HE staining or Masson’s trichrome assay. (**B**) Bleomycin-induced mouse model of lung fibrosis was established as verified by H&E-staining and Masson’s trichrome assays for collagen deposition. (**C**) XIST expression was determined in PF tissues by using real-time PCR assays. (**D**) LV-sh-XIST was used to achieve XIST knockdown in PF mice, as verified by using real-time PCR assays. (**E**) sh-XIST was used to establish XIST inhibition in BLM-induced mouse PF. The morphologic changes were exhibited by HE staining and Masson’s trichrome assays. (**F**) The protein levels of β-catenin, Collagen I and α-SMA in response to XIST knockdown in PF tissues were determined by using Western blot assays. The data are showed as mean ± SD of three independent experiments. ** *P* < 0.01.

BLM-induced mouse model of PF was established according to previous study [21]. A total of 36 mice were involved; a group of 12 mice were injected with saline (control), sacrificed on day 28 after injection, lung tissues obtained for RNA extraction, HE staining or Masson’s trichrome assay; a group of 24 mice were injected with BLM, 12 of them sacrificed on day 28, the rest 12 of them were infected with LV-sh-NC (control) or LV-sh-XIST on day 28, sacrificed 4 more days later for RNA extraction, HE staining or Masson’s trichrome assay (Figure 1A). Lung tissues of BLM-induced mouse model of PF were verified by HE staining and Masson’s trichrome assay (Figure 1B). XIST expression in PF mice and control group was determined by using PCR assays. Results showed that XIST was significantly upregulated in lung tissues of PF mice (Figure 1C). In lung tissues of BLM-induced PF mice infected with LV-sh-XIST, XIST expression was reduced compared with LV-sh-NC group (Figure 1D). As showed by HE staining and Masson’s trichrome assay, the mice treated with LV-sh-XIST exhibited decreased PF morphology (Figure 1E). To confirm the effect of XIST knockdown on PF mice, the protein levels of β-catenin, an essential faction of Wnt/β-catenin signaling, and EMC marker proteins, Collagen I and α-SMA. Results from Western blot assays showed that protein levels of β-catenin, Collagen I and α-SMA in tissues of LV-sh-XIST-infected PF mice were significantly reduced compared with LV-sh-NC group (Figure 1F), suggesting the suppressive role of XIST knockdown in BLM-induced PF.

### XIST knockdown suppressed human and mouse fibroblast proliferation

**Figure 2:**
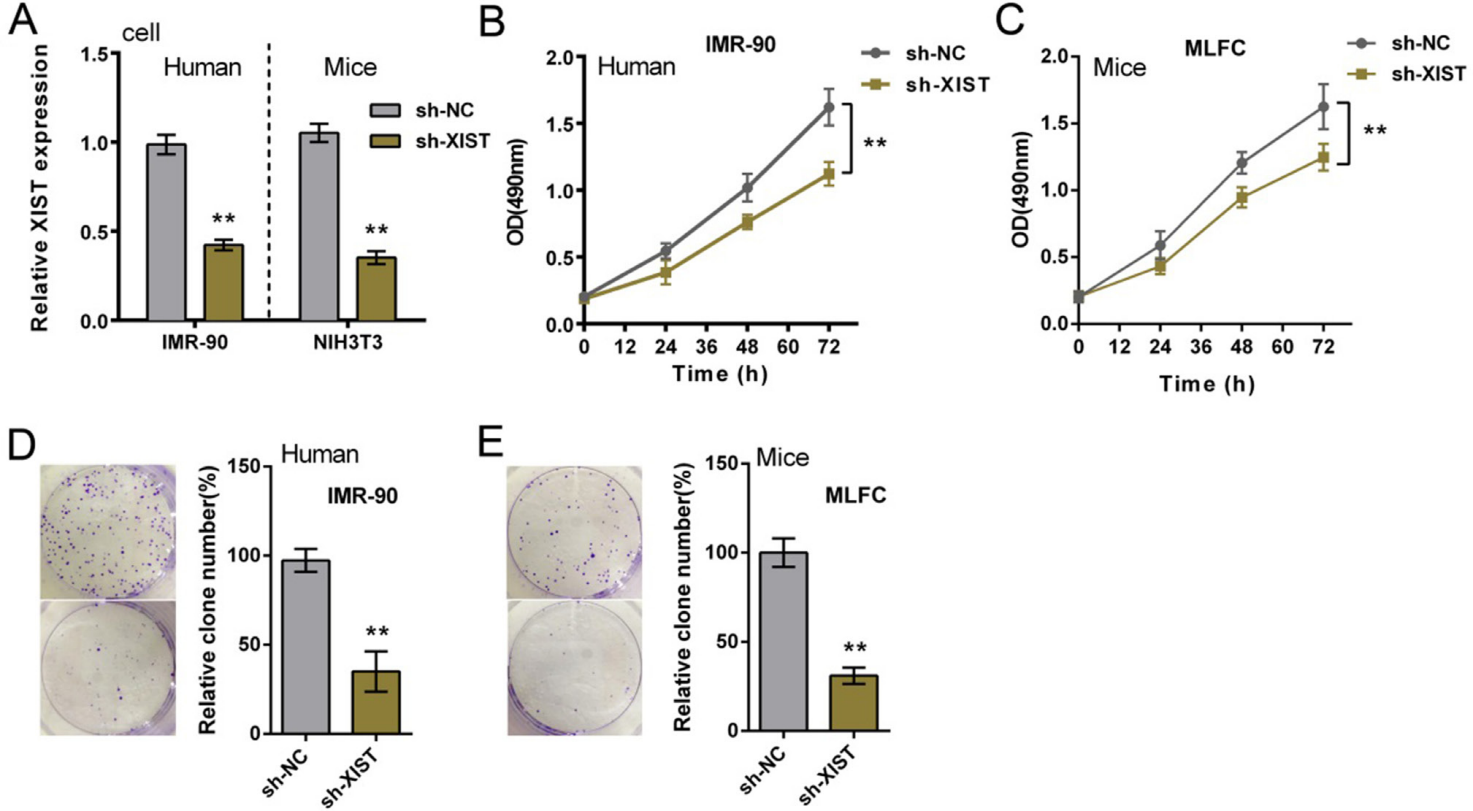
XIST knockdown suppressed human and mouse fibroblast proliferation (**A**) sh-XIST was introduced into IMR-90 and MLFCs to achieve XIST knockdown, as verified by using real-time PCR assays. (**B**) and (**C**) the proliferation of IMR-90 and MLFCs in response to XIST knockdown was determined by using MTT assays. (**D**) and (**E**) the colony formative capacity of IMR-90 and MLFCs in response to XIST knockdown was determined by using Colony formation assays. The data are showed as mean ± SD of three independent experiments. **P < 0.01.

Since PF is commonly considered the result of a recurrent injury to the alveolar epithelium followed by an uncontrolled proliferation of fibroblasts [22], next we investigated the function of XIST in fibroblasts proliferation. Human fibroblast, IMR-90, and primary mouse fibroblast were infected with sh-NC (control) of sh-XIST, the inhibitory efficiency of sh-XIST was verified by using real-time PCR assays (Figure 2A). As exhibited by MTT assays, after sh-XIST-induced XIST knockdown, the proliferation of IMR-90 and MLFCs was significantly suppressed, compared with sh-NC group (Figure 2B and 2C). Colony formation assays obtained similar results: XIST knockdown suppressed IMR-90 and MLFCs colony formative capacity (Figure 2D and 2E). These data indicated that XIST knockdown suppressed human and mouse fibroblast proliferation.

### XIST knockdown inhibited human and mouse fibroblast ECM

**Figure 3:**
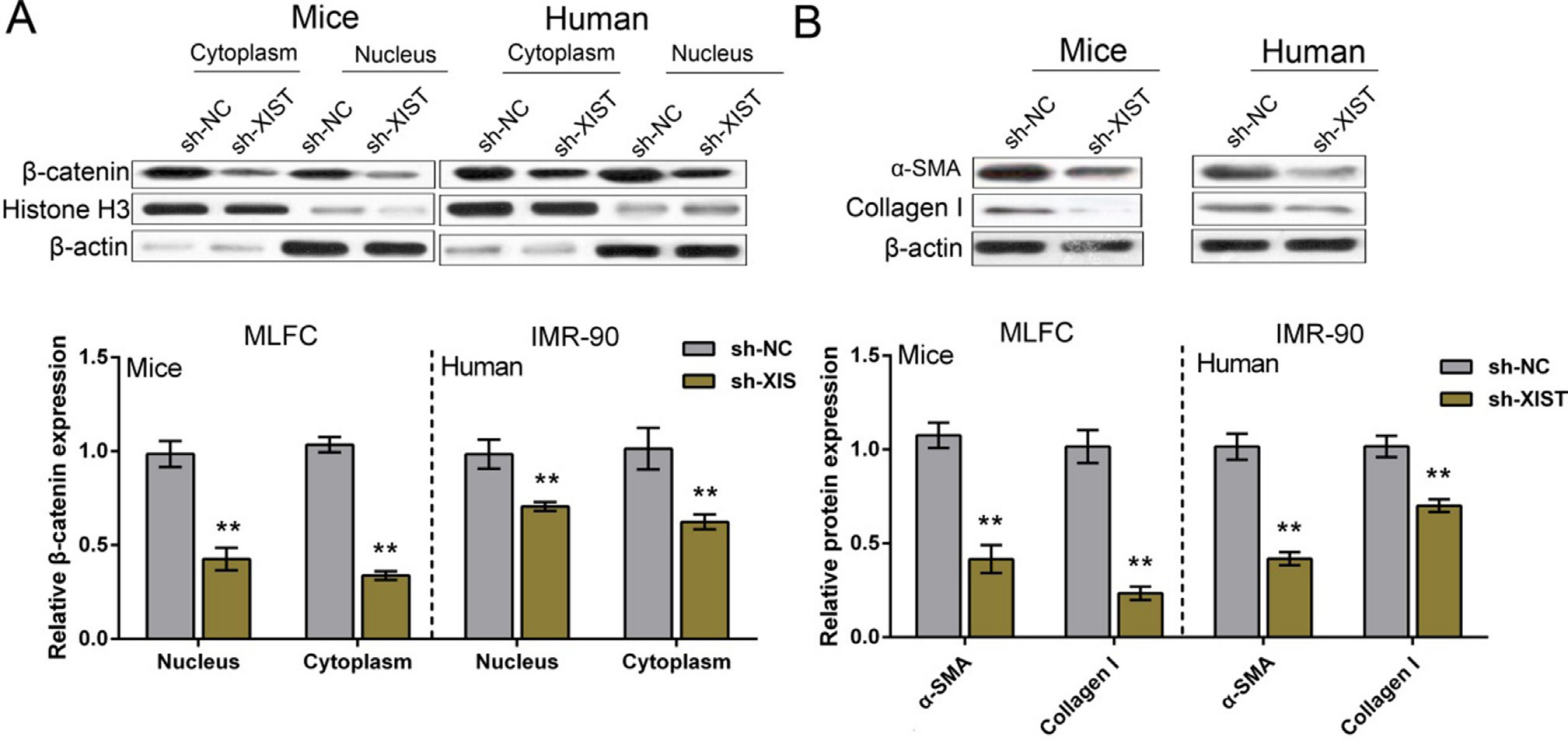
XIST knockdown inhibited human and mouse fibroblast ECM (**A**) After XIST knockdown, the protein levels of β-catenin in nucleus and cytoplasm of IMR-90 and MLFCs were determined by using Western blot assays. (**B**) After XIST knockdown, the protein levels of ECM factor, Collagen I and α-SMA, in IMR-90 and MLFCs were determined by using Western blot assays. The data are showed as mean ± SD of three independent experiments. ** *P* < 0.01.

We have revealed that XIST knockdown reduced β-catenin and ECM protein levels in PF mice tissues; next we determined β-catenin and ECM protein levels in IMR-90 and MLFCs. β-catenin protein levels in nucleus (compared to Histone H3) and cytoplasm (compared to β-actin) of IMR-90 and MLFCs were determined by using Western blot assays; results showed that after XIST knockdown, β-catenin protein levels were significantly reduced in either nucleus (compared to Histone H3) or cytoplasm (compared to β-actin) of IMR-90 and MLFCs (Figure 3A). Consistent with the results of tissue experiments, XIST knockdown also reduced the expression of ECM proteins, Collagen I and α-SMA (Figure 3B).

### XIST competed with β-catenin for miR-139 binding in IMR-90 and MLFCs

**Figure 4:**
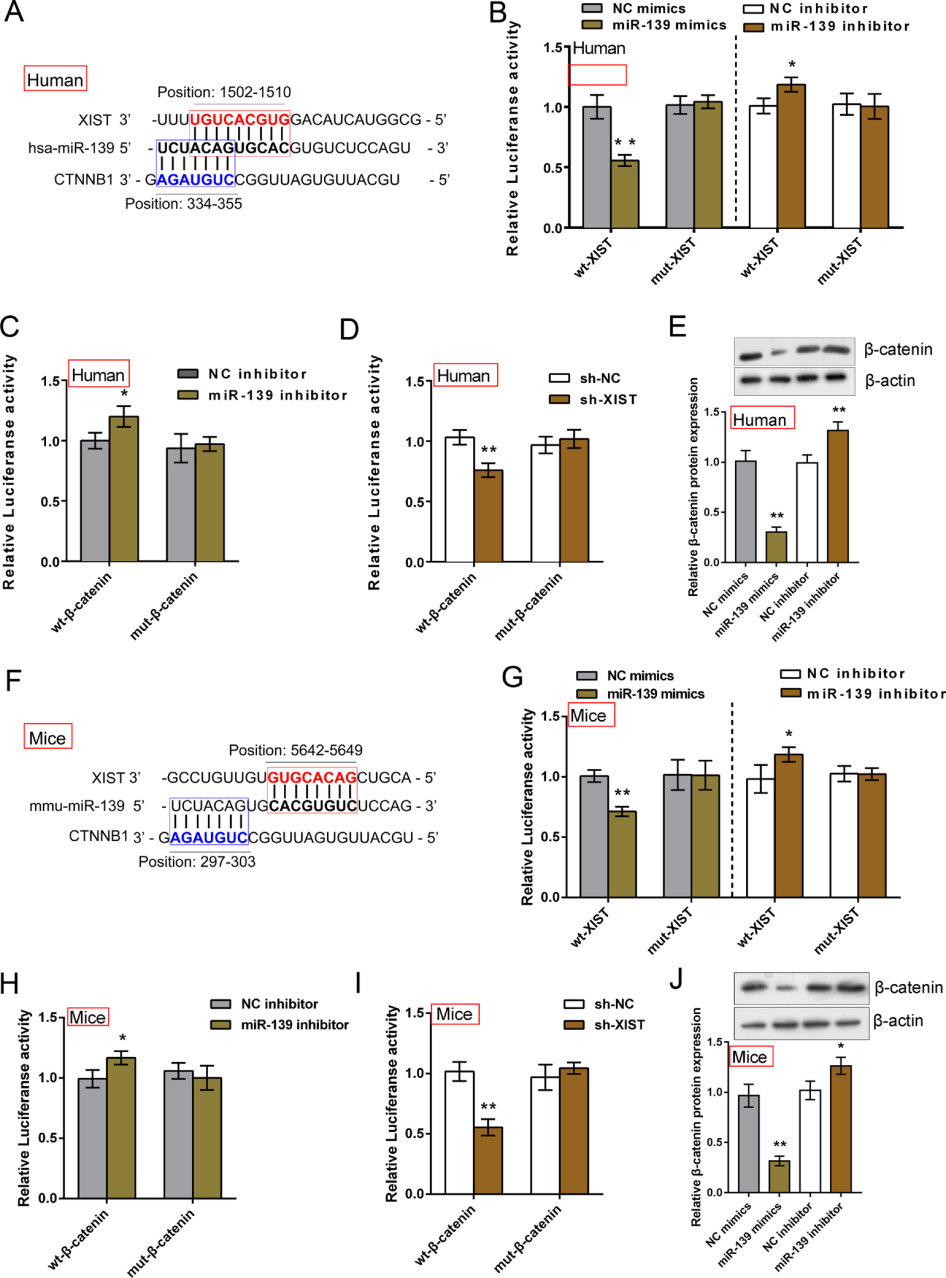
XIST competed with β-catenin for miR-139 binding in IMR-90 and MLFCs (**A**) and (**F**) Online tools predicted that XIST and β-catenin shared an almost identical binding site of miR-139. A wt-XIST luciferase reporter gene vector (human or mice), a mut-XIST vector containing a 7 bp (human) or a 6 bp (mice) mutation on putative binding site of miR-139, a wt-β-catenin vector (human or mice), a mut-β-catenin vector containing a 5 bp mutation on putative binding site of miR-139 (human or mice) was constructed. (**B**) The indicated XIST vectors (human) were co-introduced into IMR-90 cells with miR-139 mimics or miR-139 inhibitor; the luciferase activity was determined by using dual luciferase assays. (**C**) The indicated β-catenin vectors (human) were co-introduced into IMR-90 cells with miR-139 inhibitor; the luciferase activity was determined by using dual luciferase assays. (**D**) The indicated β-catenin vectors (human) were co-introduced into IMR-90 cells with sh-XIST; the luciferase activity was determined by using dual luciferase assays. (**E**) The protein levels of β-catenin in miR-139 mimics- or miR-139 inhibitor-transfected IMR-90 cells were evaluated using Western blot assays. (**G**) The indicated XIST vectors (mice) were co-introduced into MLFCs with miR-139 mimics or miR-139 inhibitor; the luciferase activity was determined by using dual luciferase assays. (**H**) The indicated β-catenin vectors (mice) were co-introduced into MLFCs with miR-139 inhibitor; the luciferase activity was determined by using dual luciferase assays. (**I**) The indicated β-catenin vectors (mice) were co-introduced into MLFCs with sh-XIST; the luciferase activity was determined by using dual luciferase assays. (**J**) The protein levels of β-catenin in miR-139 mimics- or miR-139 inhibitor-transfected MLFCs were evaluated using Western blot assays. The data are showed as mean ± SD of three independent experiments. *P < 0.05, **P < 0.01.

LncRNAs can serve as a ‘sponge’ to microRNAs and prevent them from binding to mRNAs based on the competing endogenous RNA (ceRNA) hypothesis [14]. We found that XIST shared an almost identical binding site of miR-139 with β-catenin by using online tools (Figure 4A and 4F). To further investigate whether XIST competes with β-catenin for miR-139 binding, luciferase assays were performed. A wt-XIST luciferase reporter gene vector, a mut-XIST vector containing a 7 bp (human) or 6 bp (mice) mutation on putative binding site of miR-139, a wt-β-catenin vector, a mut-β-catenin vector containing a 5 bp mutation on putative binding site of miR-139 was constructed (Figure 4A and 4F). The indicated vectors were co-transfected into IMR-90 or MLFCs with miR-139 mimics, miR-139 inhibitor or sh-XIST, respectively; the luciferase activity was then determined by using dual luciferase assays. Results showed that in IMR-90 cells, the luciferase activity of wt-XIST vectors was significantly suppressed by miR-139 mimics, promoted by miR-139 inhibitor; after mutation on the putative binding site of miR-139 in XIST, the effect of miR-139 mimics or miR-139 inhibitor on luciferase activity was abolished (Figure 4B). Similarly, the luciferase activity of wt-β-catenin was promoted by miR-139 inhibitor; the effect of miR-139 inhibitor was abolished after mutation on the putative binding site (Figure 4C). Moreover, when co-transfected with sh-XIST and wt-β-catenin/mut-β-catenin, similar results as co-transfecting miR-139 mimics and wt-β-catenin/mut-β-catenin were observed: luciferase activity of wt vector was suppressed by sh-XIST; sh-XIST-induced suppression of luciferase activity was abolished by mutation on binding site (Figure 4D), suggesting sh-XIST exerts similar function as miR-139 mimics. Further, β-catenin protein levels in response to miR-139 mimics and miR-139 inhibitor in IMR-90 cells were evaluated using Western blot assays. Results showed that miR-139 negatively regulated β-catenin protein levels (Figure 4E). In MLFCs, almost identical results were observed (Figure 4G, 4H and 4I). We also evaluated β-catenin protein levels in MLFCs; similar results were observed (Figure 4J). These data indicated that miR-139 directly binds to XIST and β-catenin, respectively; XIST competes with β-catenin for miR-139 binding.

### The functional role of miR-139 in XIST regulating β-catenin and ECM proteins

**Figure 5:**
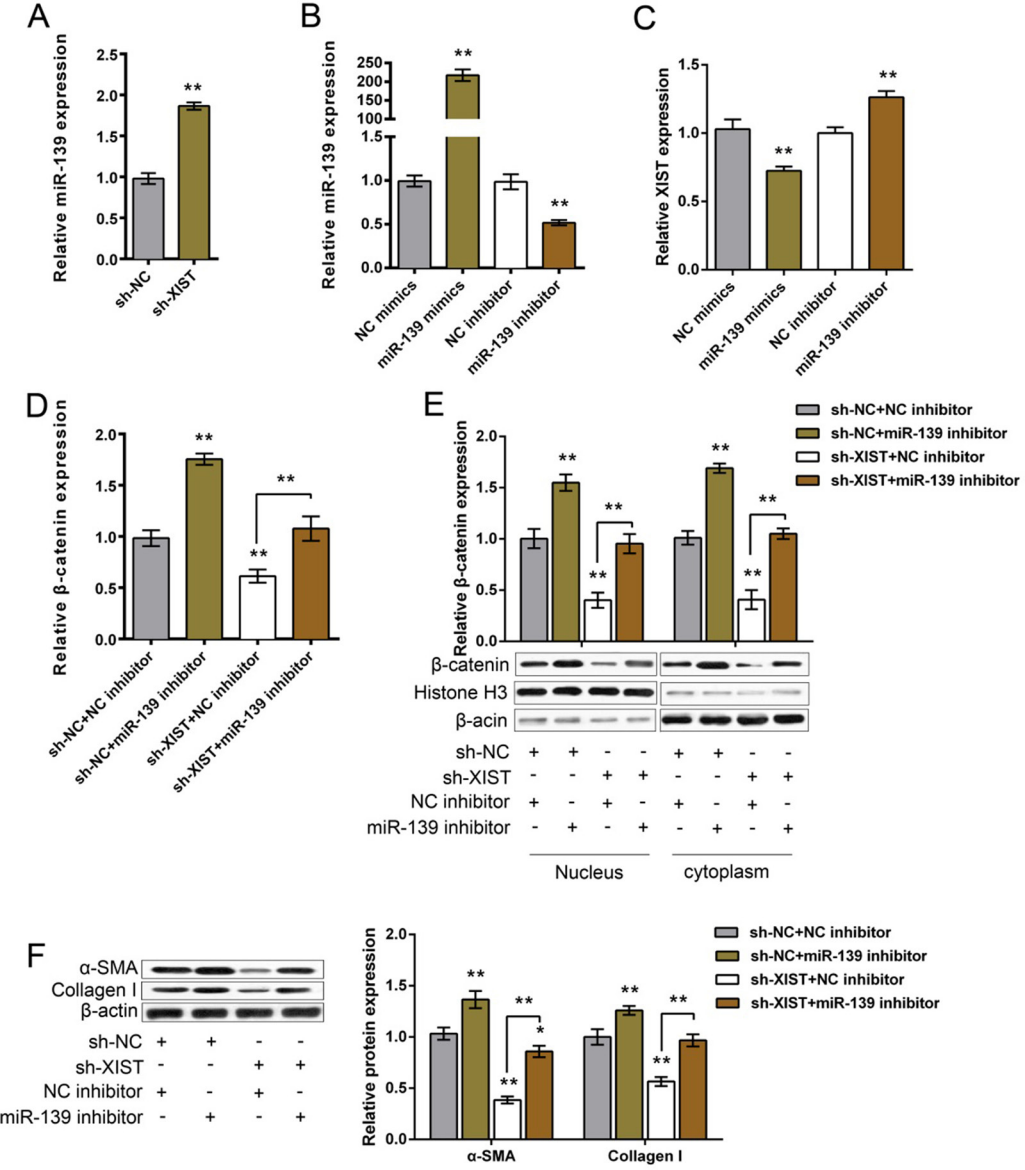
The functional role of miR-139 in XIST regulating β-catenin and ECM proteins (**A**) miR-139 expression in response to XIST knockdown in MLFCs was determined by using real-time PCR. (**B**) miR-139 mimics or miR-139 inhibitor was transfected into MLFCs to achieve ectopic miR-139 expression or miR-139 inhibition, as verified by real-time PCR assays. (**C**) The expression levels of XIST in response to ectopic miR-139 expression or miR-139 inhibition in MLFCs was determined by using real-time PCR assays. (**D**) and (**E**) After co-processing sh-XIST and miR-139 inhibition in MLFCs, β-catenin protein levels (total, nucleus, cytoplasm) were determined by using Western blot assays. (**F**) After co-processing sh-XIST and miR-139 inhibition in MLFCs, Collagen I and α-SMA protein levels were determined by using Western blot assays. The data are showed as mean± SD of three independent experiments. *P < 0.05, **P < 0.01.

We have revealed that XIST competes with β-catenin for miR-139 binding; next we further investigated the functional role of miR-139 in XIST regulating β-catenin and ECM proteins in MLFCs. After sh-XIST-induced XIST knockdown, miR-139 expression was upregulated (Figure 5A). miR-139 mimics or miR-139 inhibitor was transfected into MLFCs to achieve ectopic miR-139 expression or miR-139 inhibition, as verified by using real-time PCR assay (Figure 5B). XIST expression was upregulated by miR-139 inhibitor, whereas downregulated by ectopic miR-139 expression (Figure 5C). Next, sh-XIST and miR-139 inhibitor were co-introduced into MLFCs; β-catenin and ECM protein levels were then determined by using Western blot assays. The protein levels of β-catenin were significantly increased by miR-139 inhibitor (total, nucleus and cytoplasm), reduced by sh-XIST (total, nucleus and cytoplasm); the promotive effect of miR-139 inhibitor on β-catenin protein could be partially reversed by sh-XIST (total, nucleus and cytoplasm) (Figure 5D and 5E). Similar as β-catenin, the protein levels of Collagen I and α-SMA were increased by miR-139 inhibitor, reduced by sh-XIST; the promotive effect of miR-139 inhibitor on Collagen I and α-SMA protein levels could be partially reversed by sh-XIST (Figure 5F). These data indicated that miR-139 is involved in XIST regulating PF; miR-139 can partially reverse the effect of XIST on PF.

### Expression of miR-139 and β-catenin and their correlations with XIST in tissues

**Figure 6:**
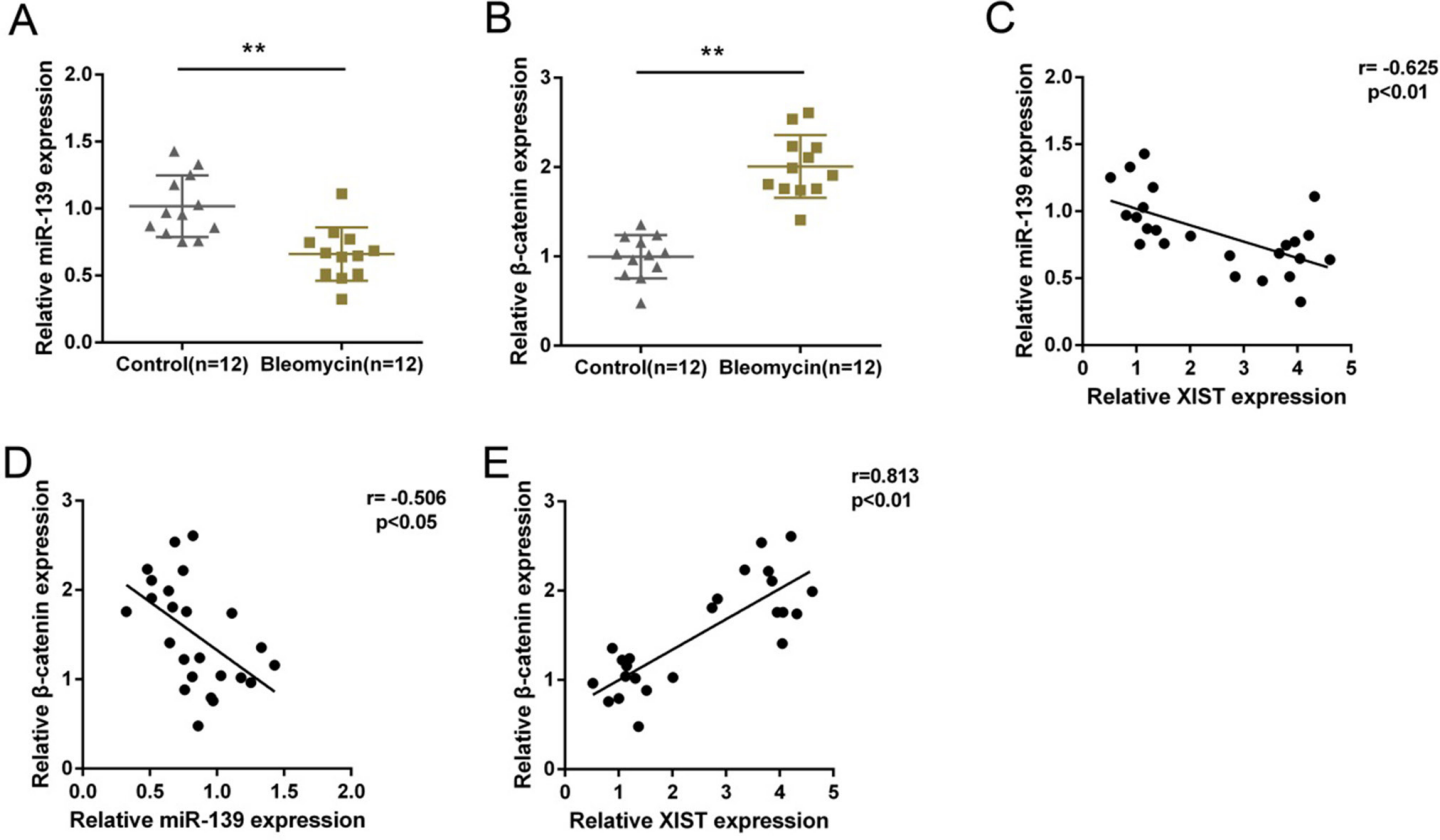
Expression of miR-139 and β-catenin and their correlations with XIST in tissues (**A**) and (**B**) miR-139 and β-catenin expression in PF and normal tissues were determined by using real-time PCR assays. The data are showed as mean ± SD of three independent experiments. **P < 0.01. (**C**, **D**) and (**E**) The correlation between XIST and miR-139, between β-catenin and miR-139, between β-catenin and XIST was analyzed by using Spearman’s rank correlation analysis.

MiR-139 and β-catenin expression in PF and normal tissues were determined by using real-time PCR assays. Results showed that in PF tissues, miR-139 was downregulated, whereas β-catenin was upregulated (Figure 6A and 6B). By performing Spearman’s rank correlation analysis, we observed an inverse correlation between miR-139 and XIST, between miR-139 and β-catenin, a positive correlation between XIST and β-catenin (Figure 6C, 6D and 6E).

## DISCUSSION

Recently, more and more studies demonstrated that PF could be regulated by lncRNAs [8]. Tang et al. revealed the promotive effect of XIST on BLM-induced PF through interaction with miR-29b [21]. In this study, we found that XIST expression was upregulated in BLM-induced PF mice, which was consistent with the previous study indicating that XIST expression was upregulated in the cystic fibrosis bronchial epithelium [11]; we further revealed that the mice treated with sh-XIST exhibited decreased PF. XIST knockdown also reduced the protein levels of ECM-related factors, Collagen I and α-SMA. In addition, XIST knockdown significantly inhibited the proliferation of human and mouse fibroblast, IMR-90 and MLFCs, as well as the protein levels of β-catenin, Collagen I and α-SMA in IMR-90 and MLFCs, suggesting the suppressive role of XIST knockdown in PF progression. However, the mechanism by which XIST regulates PF remains unclear.

LncRNAs may regulate gene expression in many ways during pulmonary fibrosis. New studies showed that lncRNAs act as key ceRNAs [14, 23, 24], thereby greatly enhancing the functionality of lncRNAs. In the present study, we demonstrated that XIST inversely regulated miR-139 expression by directly binding; in addition, β-catenin, an essential factor in Wnt/β-catenin signaling which has been reported to play a promotive role in PF [25–27], could directly bind to miR-139 on an almost identical binding site. These data suggested that XIST might compete with β-catenin for miR-139 binding during PF progression so as to repress the inhibitory effect of miR-139 on β-catenin, which inspired us to further investigate the detailed function of miR-139 in PF.

After co-introducing sh-XIST and miR-139 inhibitor into MLFCs, β-catenin, Collagen I and α-SMA protein levels were monitored. In response to XIST knockdown, β-catenin in nucleus and cytoplasm, Collagen I and α-SMA protein levels were reduced; in response to miR-139 inhibition, the indicated protein levels were increased. Moreover, miR-139 inhibition partially reversed the effect of XIST knockdown on the indicated protein levels. These all indicated that miR-139 might play a role in PF; XIST possibly exerts its function in promoting PF through competing with β-catenin for miR-139 binding.

Further, miR-139 expression was downregulated, whereas β-catenin expression was upregulated in PF tissues. Moreover, miR-139 expression was inversely correlated with XIST and β-catenin, respectively; XIST and β-catenin was positively correlated, further indicating the positive role of XIST in PF, and that XIST competes with β-catenin for miR-139 binding to promote PF progression.

In conclusion, we revealed the promotive effect of XIST on BLM-induced PF, and demonstrated the mechanism by which XIST exerts its effect on promoting human and mouse fibroblast proliferation, ECM protein expression and pulmonary fibrosis. The present study provided a potential therapy to treat PF. However, the mechanisms by which XIST exerts its functions vary under different circumstances; other promising targets in PF treatment need further investigation.

## MATERIALS AND METHODS

### Cell line and cell transfection

Human pulmonary fibroblast, IMR-90 (ATCC, USA) and mouse pulmonary fibroblast, NIH3T3 (ATCC, USA) were cultured in DMEM medium (GIBCO, USA) supplemented with 10% fetal calf serum (GIBCO, USA), 100 units/ml penicillin and 100 μg/ml streptomycin in a humidified incubator with 5% CO_2_ at 37°C.

### Animals and induction of idiopathic pulmonary fibrosis

All animal procedures were conducted in accordance with humane animal care standards approved by the Hunan Cancer Hospital Medicine Ethics Committee (Changsha, China). All experiments were performed in accordance with relevant guidelines and regulations. Male C57BL/6 mice (6–8 weeks of age; 18–20 g) were obtained from SLAC China (Shanghai, China) and maintained under specific pathogen-free conditions. The animals were acclimated to the environment for 1 week prior to treatment.

The mice were administered with BLM (Nippon Kayaku, Tokyo, Japan) intratracheally at a dose of 1.5 U/kg dissolved in a total of 0.05 ml of sterile saline. The control groups were treated with 0.05 ml of sterile saline using the same method. The mice were sacrificed on day 28 or 32 (*n* = 12 for each time point). The lung tissues were collected for RNA extraction, HE staining or Masson’s trichrome assay.

### Lentivirus production and infection

To generate XIST knocked-out or control lentivirus, the pLVX vector containing shRNA XIST or the control sequence were co-transfected into 293 cells together with the plasmids pHelper1.0 and pHelper2.0 (Genechem) that contain the necessary elements for virus packaging. The transfection was generated using Lipofectamine 2000 (Invitrogen) following the manufacturer’s instructions. Supernatants containing the lentivirus were harvested at 72 h, and then concentrated with Lenti-Pac^™^ Lentivirus Concentration Kit (GeneCopoeia), the viral titers of the lentivirus were determined before infection. HCNSLCs cells were plated at a concentration of 2 × 10^6^ cells/ml, incubated for 16 h. For infection, 1.5 ml/well viral supernatant was used to replace the medium. Cells were incubated at 37°C for 10 h, and then the fresh media was used to replace the viral supernatant. 48 h after the infection, 2 mg/ml puromycin was used to select cells. 5 days later, the infection efficiency was verified using Western blot or real-time PCR assays.

28 days after intratracheal treatment of BLM, lentivirus shRNA XIST or the control sequence were dissolved in saline (10 mg/ml, 0.5 ml). The lentivirus vectors were freshly prepared for the experiments. The lentivirus with shRNA XIST or the control sequence was then injected into PF mice tail. 4 more days later, 12 mice were sacrificed and the lung tissues were collected for RNA extraction, HE staining or Masson’s trichrome assay.

### H&E and Masson’s staining

Lung sections were stained with H&E and Masson’s trichrome and a quantifying assayication of Masson’s trichrome, respectively. Briefly, the severity of fibrosis was graded and scored on a scale of 0–8. Ten fields per section at ×200 magnifications were randomly selected per mouse, and two blinded pathologists carefully and independently examined 60 fields per group using Nikon DS-Fi1-U2 microscope (Nikon, Tokyo, Japan).

### Cell transfection

Both the miRNA mimics and miRNA inhibitors were synthesized by Genepharma Company (Shanghai, China). Oligonucleotide and plasmid transfection were conducted by using the Lipofectamine^™^ 2000 transfection reagent (Invitrogen, USA), followed by the protocol recommended by the manufacturer. After 48 h transfection, the cells were collected and used for further investigations.

### RNA extraction and real-time PCR

TRIZOL Reagent (Invitrogen, USA) was used to extract total RNA. Then RNA sample reverse transcription was achieved by High Capacity cDNA Reverse Transcription Kit (Applied Biosystems, USA). The Fast Start Universal SYBR Green Master (Roche, USA) was applied for the quantitative RT-PCR. 2^−ΔΔCT^ method was used to analyze the relative fold changes.

### Western blot

Cell lysates were lysed by RIPA buffer (Sigma-Aldrich, USA) with Complete Protease Inhibitor Cocktail (Roche, USA). Cell lysates were transferred to 1.5 mL tube and kept at −20°C before use. SDS-PAGE was conducted to separate the cellular proteins. And all the cellular proteins within this study were separated by 5% stacking gel and 10% running gel. The molecular weight of candidate proteins was referred to the information of the Pre-stained SeeBlue rainbow marker (Invitrogen, USA) loaded in parallel. The membranes were probed with the following antibodies: β-catenin (Cat# E247, Abcam, USA), Collagen I (ab34710, Abcam), α-SMA (ab5694, Abcam), Histone H3 (ab1791, Abcam), β-actin (Cat# ACTN05 (C4), Abcam) and GAPDH (Cat# 6C5, Abcam). The blots were detected on Kodak film developer (Fujifilm, Japan).

### Cell proliferation assay

Cell proliferation was measured by 3-(4, 5-Dimethylthiazol-2-yl)-2, 5-diphenyltetrazolium bromide (MTT) assay. IMR-90 and MLFCs (2.5 × 10^4^/well) were seeded in 96-well plates in triplicate. The cells were serum-starved the following day and infected with 50 nM of either the sh-XIST or the sh-NC controls. The MTT (Sigma, USA) was added to a final concentration of 0.5 mg/ml, and the cells were incubated for 4 h at 37°C. The absorbance at 490 nm was measured by micro plate reader (Bio-rad, USA). Each experiment was repeated at least three times.

### Soft agar assay

IMR-90 and MLFCs treated with sh-NC or sh-XIST (10 ng/ ml) were mixed into 0.5 ml of 0.35% agar containing growth medium and layered over a base of 0.5% agar to prevent anchorage-dependent cell growth. Once this layer was solidified, it was overlaid with 1 ml of normal growth medium, which was replaced every 2 days for 14 days. A colony is defined as a cell aggregate larger than 100 mm. Pictures were taken and visible colonies were counted after 14 and 28 days.

### Luciferase reporter assay

IMR-90 and MLFCs were seeded into a 24-well plate. After cultured overnight, cells were co-transfected with the wild-type and mutated XIST or wild-type and mutated β-catenin 3′UTR reporter plasmid or pRL-TK plasmids and miR-139 mimics or miR-139 inhibitor. Luciferase assays were performed 48 h after transfection using the Dual Luciferase Reporter Assay System (Promega, WI, USA).

### Statistical analysis

The data are presented as mean values, and the error bars indicate standard deviation (SD). The quantitative variables between two groups were compared using the independent-samples t test, and among more groups via one-way analysis of variance (ANOVA) using Dunnett’s test (day 0 as the control group). *P* < 0.05 was considered statistically significant.
